# Surveillance and vaccine effectiveness of pertussis, the Netherlands, 2012 to 2024, with an unprecedented surge in 2023 and 2024

**DOI:** 10.2807/1560-7917.ES.2026.31.15.2500919

**Published:** 2026-04-16

**Authors:** Annika van Roon, Dimphey van Meijeren, Brechje de Gier, Margreet te Wierik, Hester de Melker

**Affiliations:** 1 National Institute for Public Health and the Environment (RIVM), Bilthoven, the Netherlands

**Keywords:** Whooping cough, Pertussis, Disease outbreaks, Surveillance, Vaccination, Vaccine effectiveness, maternally-acquired immunity

## Abstract

**BACKGROUND:**

A surge in pertussis occurred in the Netherlands in 2023–24. Infant vaccination uptake decreased from 95% in 2011 to ca 86% in 2024. Maternal vaccination was introduced in 2019, with uptake ca 70%.

**AIM:**

To describe pertussis epidemiological trends in the Netherlands.

**METHODS:**

We conducted a retrospective study using pertussis notification data from 2012 to 2024 and estimated infant and maternal vaccine effectiveness (VE) with the screening method.

**RESULTS:**

During the COVID-19 pandemic, pertussis notifications dropped from ca 6,000 in 2013–19 to 79 in 2021 (incidence ca 35 to < 0.01/100,000 population). Notifications surged from May 2023, peaking in March 2024, resulting in 18,208 notifications in 2024 (102/100,000). Notifications and hospitalisations in 2024 were highest among infants aged 0–5 months (573 and 304/100,000) followed by infants aged 6–11 months (446 and 92/100,000). Annually, 0–2 deaths were reported; in 2023–24, 10 deaths were reported (6 infants, 4  ≥ 60-year-olds). In 2024, 83% of mothers of notified infants aged 0–2 months were unvaccinated. In 2020–24, maternal VE against pertussis in infants aged 0–2 months was 91%. In 2012–24 primary series VE was 98% at age 1, 92% at age 3, 92% post-booster at age 5, and 71% at age 9 years.

**CONCLUSION:**

Low population immunity after 2 years of reduced circulation likely contributed to the highest pertussis incidence ever recorded in the Netherlands, posing a particular threat to unprotected infants. Maternal and infant VE are high, underscoring the public health priority of enhancing vaccination uptake.

**Key public health message**

**What did you want to address in this study and why?**

Pertussis (whooping cough) is a highly contagious, vaccine-preventable respiratory infection that can cause severe illness and death especially in infants. In 2023–24, the Netherlands experienced an unusually large surge in pertussis. We wanted to understand why this occurred, who was most affected, and whether vaccines for pregnant women and children were still working well to protect against severe illness.

**What have we learnt from this study?**

In 2024, over 18,000 pertussis notifications were recorded, compared to a yearly average of 6,000, with infants under 6 months being most affected. This surge was likely caused by fewer people being naturally exposed during COVID-19, waning vaccine-derived immunity in older children, and declining vaccination rates. Maternal and childhood vaccine effectiveness remained consistently high, with no evidence of a decline during the outbreak.

**What are the implications of your findings for public health?**

Our findings confirm that both maternal and childhood pertussis vaccination are highly effective. This highlights the importance of improving vaccination uptake, especially among pregnant women and young children, to protect those most at risk. Maintaining high vaccination coverage and closely monitoring immunity gaps are essential to prevent future pertussis outbreaks and to reduce the risk of severe illness in infants.

## Introduction

Pertussis (whooping cough) is a highly contagious respiratory infection primarily caused by the bacterium *Bordetella pertussis*. Infection with other *Bordetella* species, such as *Bordetella parapertussis*, can cause pertussis-like symptoms but is usually less severe [1]. Pertussis can affect individuals of all ages, but infants (aged < 12 months) are at greatest risk of severe illness and death [2]. To prevent infants and children from becoming seriously ill because of pertussis, vaccination was introduced in the Netherlands in 1953. As a result, the number of pertussis cases and deaths decreased considerably [3]. Yet, since 1996, regular epidemics have occurred [4,5], with the most recent one taking place in 2012 [6].

Since 1957, children in the Netherlands have been offered the combined diphtheria, tetanus and pertussis vaccine as part of the national immunisation programme (NIP). The NIP is funded and organised by the government, providing free-of-charge vaccinations for all children in the Netherlands. Vaccinations are administered at the well-baby clinics operated by the Centres for Youth and Family (CJG) and the Municipal Public Health Services. Initially, children received four doses of the whole cell pertussis vaccine (DTwP) during their first year of life, then in 2001, an acellular pertussis booster vaccination at 4 years of age was added to the NIP. In 2005, the whole-cell pertussis component in the infant vaccinations was replaced with an acellular pertussis component (DTaP-IPV-Hib) [7]. Maas et al. showed that for children eligible for vaccination, pertussis infection rates in the Netherlands decreased between 2003 and 2010, but infection rates had increased in adolescents and adults and remained high in young infants [6]. To protect these infants too young to be (fully) vaccinated, maternal pertussis vaccination (Tdap) from 22 weeks of pregnancy onward was introduced in the NIP on 16 December 2019. In the 2024 vaccination schedule, infants were offered three primary doses of the acellular pertussis vaccine at 3, 5 and 11 months of age if their mothers received a maternal pertussis vaccination, followed by a booster vaccination at 4 years of age [7]. Infants born to mothers who were not vaccinated against pertussis during pregnancy, as well as certain high-risk groups, such as premature infants, were offered an additional dose at 2 months of age [8].

During and immediately following the COVID-19 pandemic (2020–23), the number of pertussis notifications in the Netherlands was very low. However, starting in May 2023, pertussis notifications in the Netherlands began to increase, surging to unprecedentedly high levels in 2024. The aim of this paper is to describe the epidemiological trends from 2012 onward, including the surge of pertussis in the Netherlands in 2023–24. We analysed pertussis notifications, including hospitalisations and deaths, by age and vaccination status, and evaluated the vaccine effectiveness of maternal and childhood vaccination in the Netherlands.

## Methods

### Study design and setting

We conducted a retrospective observational study of all notifications of pertussis from 2012 to 2024 in the Netherlands.

Since 1976, pertussis is a notifiable disease in the Netherlands. Notifications that meet the case definition (see below) are registered by the municipal health service and reported pseudonymised to the National Institute for Public Health and the Environment (RIVM). All notifications recorded in the national registration Osiris database up to 14 August 2025, with disease onset between 1 January 2012 and 31 December 2024, were included in this study. These records include information such as the year and month of birth (the latter only for children less than 2 years of age), date of symptom onset, hospitalisation and mortality information, vaccination status (only collected for children under 12 years of age), and maternal vaccination status for infants (from 1 April 2020 onward). 

### Case definitions

The notification criteria for pertussis are as follows [9]: children or adults with cough symptoms lasting at least 14 days or individuals exhibiting one of the following three symptoms: (i) paroxysmal coughing, (ii) coughing with a whooping sound during inhalation, or (iii) vomiting after coughing. Additionally, cases must meet at least one of the following laboratory criteria or have had recent contact (within 3 weeks) with a person in whom the infection has been laboratory confirmed: (i) detection of *B. pertussis* or *B. parapertussis,* or (ii) a high antibody titre in the first serum sample consistent with a recent *B. pertussis* infection, i.e. > 100 IU/ml, or (iii) at least a three-fold titre increase in two-point serology consistent with a recent *B. pertussis* infection.

Individuals diagnosed by PCR in an early stage of disease and (therefore) not (yet) meeting symptom criteria should also be notified. In this case, the notifying municipal health service should describe why the case was notified although not meeting the notification criteria which is subsequently reviewed by the RIVM. This includes, for instance, infants or young children with atypical symptoms, individuals with cough symptoms less than 14 days or individuals at high risk of severe disease who have had recent contact with a person with a confirmed infection. All notifications with a valid reason are included in this study.

### Vaccination status

Vaccination status of the child is obtained from the parents or retrieved from the national vaccination registry Praeventis [10], which is managed by the RIVM. Maternal vaccination status is obtained from the mother or retrieved from Praeventis when the mother cannot be reached. However, from 1 January 2022 onward, informed consent was required, and parents needed to give permission for vaccination data sharing with the RIVM. Therefore, a missing vaccination status indicates that the child or mother was not vaccinated or that no informed consent was given. The percentage of vaccinations recorded but without informed consent varies over time, by region and by vaccine type. In 2024, this percentage was 3% of all vaccinations administered within the NIP, with lower percentages for the primary pertussis series (2%), compared with the booster (8%) and maternal vaccination (10%) [11]. Mortality data (International Classification of Disease number 10 (ICD-10) [12] codes: A37.0, A37.1, A37.9) up to and including 2023 were available from Statistics the Netherlands (CBS). Data for 2024 contain mortality as reported in the data extracted from Osiris at the time of notification.

National vaccination coverage data as reported in the annual National Vaccination Coverage Report [13] were used and included national coverage estimates for the primary vaccine series, booster doses and maternal vaccination, by birth cohort, municipality and year.

Vaccination status (fully vaccinated, partially vaccinated, unvaccinated or unknown) of each notified case was determined according to their age and the recommended vaccination schedule for their birth year, as per the NIP (Table 1).

**Table 1:** Overview of pertussis vaccination within the national immunisation programme per birth year in the Netherlands, 1954–2024

Notified cases	National immunisation programme schedule
Born before 1954	No NIP
Born between 1954–1997	Primary series at 2–3–4–11 months of age
Born between 1998–2019^a^	Primary series at 2–3–4–11 months + booster at 4 years of age
Born in 2020^b^	Maternal vaccination + primary series at 3–5–11 months + booster at 4 years of age, or Primary series at 2–3–5–11 months + booster at 4 years of age
Born between 2021–2023	Maternal vaccination + primary series at 3–5–11 months + booster at 5 years of age, or Primary series at 2–3–5–11 months + booster at 5 years of age
Born from 2024 onwards	Maternal vaccination + primary series at 3–5–12 months + booster at 5 years of age, or Primary series at 2–3–5–12 months + booster at 5 years of age

NIP: national immunisation programme.

^a^ Up to 2001, children received a whole cell pertussis vaccine (DTwP) during their first year of life. In 2001, an acellular pertussis booster vaccination at 4 years of age was added to the NIP. In 2005, the whole-cell pertussis component in the infant vaccinations was replaced with an acellular pertussis component (DTaP-IPV-Hib) [7].

^b^ Maternal vaccination (Tdap) was included for infants born from 1 April 2020 onwards, which marks almost 4 months post-introduction of maternal vaccination in the Netherlands. All mothers of 0–2-month-old infants were eligible for vaccination.

### Vaccine effectiveness

Vaccine effectiveness against notifications of infection and hospitalisation was estimated using the screening method, comparing the proportion of vaccinated cases (PCV) to the proportion of vaccinated individuals in the total population (PPV), stratified by birth cohort, using the equation: VE = 1−(PCV/1−PCV)*(1−PPV/PPV) [14]. Vaccine effectiveness against death was not calculated because of small numbers. The PPV estimates were sourced from the National Vaccination Coverage Report [13] and are provided in Supplementary Table S1. For the primary series and booster dose, the most recent available coverage for the relevant birth cohorts was used. However, because of the introduction of informed consent from 1 January 2022 onwards, PPV is underestimated and leads to underestimation of VE [15]. A sensitivity analysis was performed in which the vaccination coverage of the affected birth cohorts —namely, 2021–22 for the primary series and 2018–19 for the booster dose — was increased by 3 and 8 percentage points, respectively. These are national overall estimates for the percentage of parents that did not give informed consent.

For the VE of maternal vaccination, cases aged 0–2 months with disease onset between 1 April 2020 (almost 4 months after introduction of the maternal vaccination in the Netherlands and therefore all mothers of 0–2-month-old infants were eligible for vaccination) and 31 December 2024 and with known maternal vaccination status were included. For the primary series and booster VE, cases with disease onset from 2012 to 2024 were included. For booster VE, analysis was limited to cohorts born from 2005 onwards to ensure the inclusion of only birth cohorts that were eligible for primary vaccination with the acellular pertussis vaccine.

For maternal vaccination, a PPV of 70% was used [16]. This represents the coverage determined for 2020, before the introduction of the informed consent procedure. Supplementary Table S1 provides an overview of national vaccination coverage estimates per birth cohort. However, due to uncertainty in the estimation, a sensitivity analysis was performed using a PPV estimate of 64%, which is the lowest estimate reported in recent years [13].

To ensure comparability between PPV and PCV, partially vaccinated and people with unknown vaccination status were included in the denominator when calculating PCV for the primary vaccine series and booster dose. However, a sensitivity analysis was performed in which all cases with unknown vaccination status were classified as vaccinated, to obtain the most conservative estimate. For the maternal vaccination VE, people with unknown vaccination status were excluded from the denominator of the PCV, as we used the vaccination coverage from the years before introduction of the informed consent for the PPV.

Descriptive analyses and vaccine effectiveness calculations were performed using R version 4.5.1.

## Results

**Figure 1:**
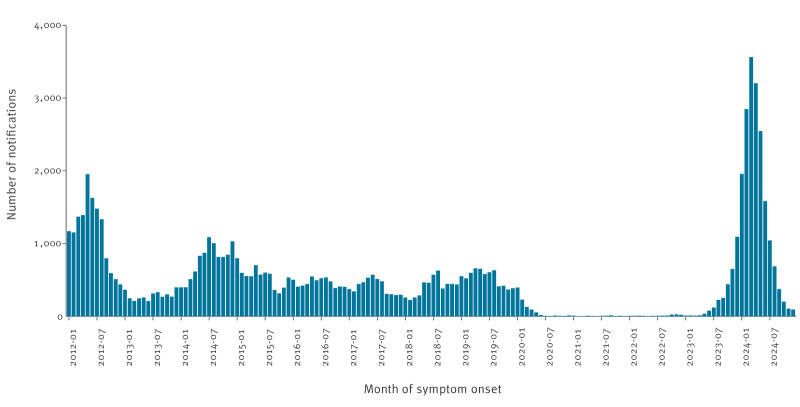
Number of pertussis notifications by month of symptom onset, the Netherlands, January 2012–December 2024 (n = 77,127)

**Figure 2:**
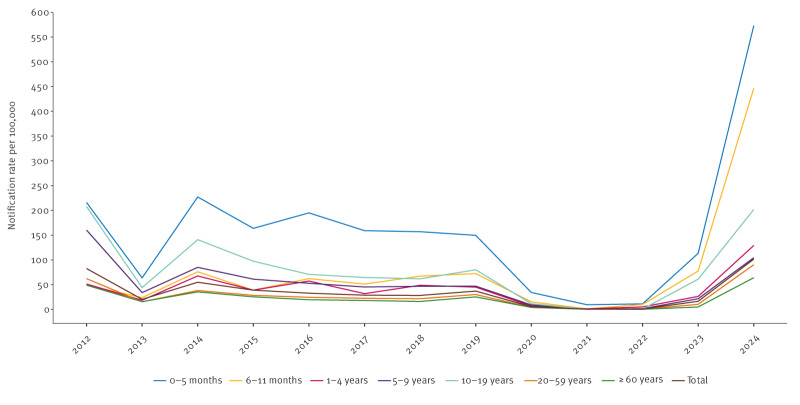
Incidence of pertussis notifications by age and year of symptom onset, the Netherlands, January 2012–December 2024 (n = 77,127)

A major peak in pertussis notifications was observed in 2012, with 13,819 notifications and an incidence of notifications of 83 cases per 100,000. Thereafter, an average of ca 6,000 notifications were recorded annually, with a smaller peak in 2014 (n = 9,231, incidence of notifications = 55/100,000), until the onset of the COVID-19 pandemic. In 2020, the number of notifications dropped to 970 with an incidence of notifications of 6 per 100,000 and declined further to 79 in 2021 and an incidence of notifications of < 0.01 per 100,000. This low level of notifications continued until early 2023. From May 2023 onwards, the number of notifications started to increase, reaching unprecedentedly high numbers in 2024 with an epidemic peak in March (n = 3,562 notifications) and a year total of 18,208 notifications (102/100,000) (Figure 1, Figure 2).

Throughout the study period, the highest incidence of notifications has consistently been observed in infants aged 0–5 months (Figure 2). Supplementary Table S2 provides the number and incidence of notifications per year and age group. Between 2012 and 2017, the age group with the second highest incidence of notifications was 10–19 years, followed by the infants aged 6–11 months. Between 2018 and 2020, the incidences of notifications of these two age groups were comparable. During the COVID-19 pandemic, the incidence of notifications was very low in all age groups. From May 2023 onwards, the incidence increased and remained highest in infants aged 0–5 months with rates of 113 per 100,000 in 2023 and 573 per 100,000 in 2024. The age groups 6–11 months and 10–19 years followed with incidences of 78 and 61 cases per 100,000 in 2023 and 446 and 202 per 100,000 in 2024, respectively. The lowest incidence of notifications was observed in individuals aged 60 years and older, at 5 per 100,000 in 2023 and 64 per 100,000 in 2024.

### Hospitalisations and deaths

**Figure 3:**
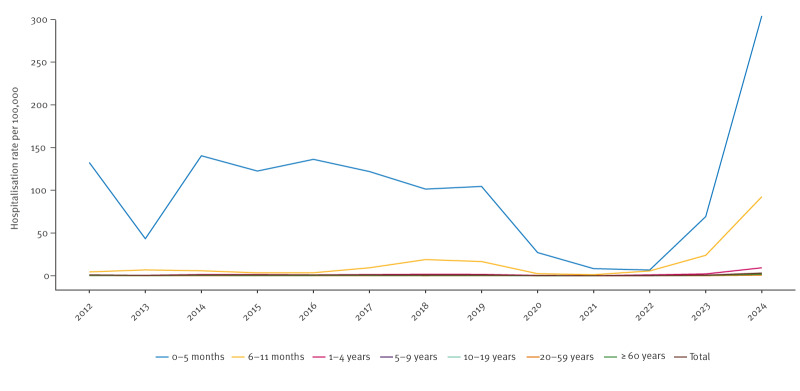
Incidence of pertussis notifications reporting hospitalisations per age and year of symptom onset, the Netherlands, January 2012–December 2024 (n = 1,901)

From 2012 to 2023, among all age groups, between 13 and 174 notifications reporting hospitalisations have been received annually with an incidence of 0–1 per 100,000. In 2024, 563 notifications reporting hospitalisations were received, with an incidence of 3 per 100,000. The highest incidence of hospitalisations has consistently been observed among infants aged 0–5 months (304/100,000), followed by those aged 6–11 months (92/100,000) (Figure 3). Supplementary Table S3 provides the number and incidence of notifications reporting hospitalisations per year and age group. In 2024, 53% (250/471) of notified infants aged 0–5 months and 21% (76/367) of those aged 6–11 months were hospitalised.

Since 2012 and up to 2022, 0–2 pertussis-related deaths were reported annually, primarily among infants. In 2023 and 2024, 10 deaths were reported: two and eight, respectively. 

Five of 10 deaths were among infants aged 0–5 months, of which three were unvaccinated including their mother. Two term infants too young to be vaccinated had mothers who received vaccination between 25 and 27 weeks. One 6–11-month-old infant was unvaccinated. Four deaths occurred in individuals aged 60 years and older.

### Vaccination status and vaccine effectiveness

#### Maternal vaccination

For 315 infants with disease onset between 2020 and 2024, maternal vaccination status was known. Supplementary Table S4 provides numbers of maternal Tdap vaccination and timeliness of vaccination. Of these, 53 (17%) had received maternal Tdap vaccination (Table 2) and 11 of these 53 (21%) were either born prematurely (n = 10) or the mother was vaccinated less than 2 weeks before birth (n = 1).

**Table 2:** Maternal Tdap vaccine effectiveness estimates against notification of infection for infants aged 0–2 months per year of symptom onset*,* the Netherlands, April 2020–December 2024 (n = 315)

Year	Number of infants with known maternal Tdap status^a^	Maternal Tdap received	VE (%)	95% CI
Overall				
2020	8	3	74	−8 to 94
2021	0	0	NA	
2022	5	3	36	−285 to 89
2023	61	5	96	90 to 98
2024	241	42	91	87 to 94
**Total**	**315**	**53**	**91**	**88 to 94**
*Bordetella pertussis*				
2020	6	2	79	−17 to 96
2021	NA			
2022	NA			
2023	56	4	97	91 to 99
2024	232	41	91	87 to 93
**Total**	**294**	**47**	**92**	**89 to 94**
*Bordetella parapertussis*				
2020	2	1	57	−585 to 97
2021^b^	NA			
2022	5	3	36	−285 to 89
2023	3	1	79	−136 to 98
2024	2	0	100	NA
**Total**	**12**	**5**	**69**	**4 to 90**

CI: confidence interval; NA: not applicable; Tdap: tetanus-diphtheria-acellular pertussis; VE: vaccine effectiveness.

^a^ Nine notifications lacked information about the *Bordetella *species and were therefore not included in the species-specific VE calculations.

^b^ Infants reported in 2021 were excluded, as they were probably all part of a pseudo-outbreak of *B. parapertussis* [31].

Between 2020 and 2024, VE was 91% (95% CI: 88–94). At vaccination coverage of 64% instead of 70%, VE was estimated at 89% (95% CI: 85–92). Annual VE estimates for 2020–22 were uncertain because of low numbers. Vaccine effectiveness was 96% (95% CI: 90–98) in 2023 and 91% (95% CI: 87–94%) in 2024. When distinguishing between notifications of infection with *B. pertussis* and *B. parapertussis*, VE against *B. pertussis* was similar, and VE against *B. parapertussis* much lower, however this was based on only 12 cases (Table 2).

The proportion of cases with unknown maternal vaccination status was higher in 2024. Sensitivity analysis showed that in the most conservative scenario (all cases with unknown status were vaccinated), VE was 86% (95% CI: 82–89) and in the most optimistic scenario (all cases with unknown status were unvaccinated), VE was 92% (95% CI: 90–94) in 2024. Supplementary Table S5 provides VE estimates per year for each scenario.

Between 2020 and 2024, VE of maternal vaccination against hospitalisation due to pertussis was estimated at 90% (95% CI: 86–93) based on 227 hospitalised cases with known maternal vaccination status, of which 42 were infants whose mothers had received maternal Tdap vaccination.

#### Primary series and booster vaccination

The percentage of notified cases up to 12 years of age that were fully vaccinated according to age varies across years and age groups. Supplementary Table S6 provides the pertussis vaccination status per year and age group. In 2023, 17% (13/75) of the notified cases aged 0 to 3 months, 16% (3/19) of cases aged 4 to 5 months, and 8% (5/60) of cases aged 6 to 11 months were fully vaccinated according to age. In 2024, these percentages were higher with 37% (109/292), 21% (22/105) and 12% (34/280), respectively.

**Figure 4:**
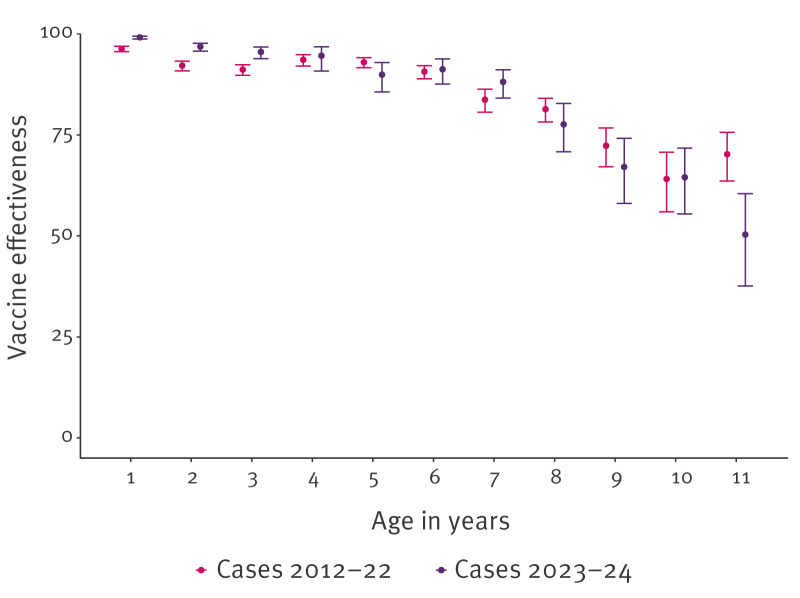
Vaccine effectiveness of the primary pertussis vaccination series and booster dose in children for notified cases, the Netherlands, 2012–2022 and 2023–2024 (n = 33,554)

The VE against notification of pertussis infection with disease onset between 2012 and 2024 was consistently high, estimated at 98% (95% CI: 97–98) at 1 year, 94% (95% CI: 93–94) at 2 years and 92% (95% CI: 91–93) at 3 years of age. Supplementary Table S7 provides VE estimates per year and age group. During the 2023–24 outbreak, VE estimates for children aged 1–3 years were higher than those for 2012–22 and the overall period (2012–24), with estimates of 99% (95% CI: 99–99), 97% (95% CI: 96–98) and 96% (95% CI: 94–97) for children aged 1, 2 and 3 years, respectively (Figure 4). Supplementary Table S7 provides VE estimates per year and age group. A sensitivity analysis with a 3-percentage-point increase in vaccination coverage for birth cohorts affected by the informed consent procedure shows small change in the VE. Supplementary Table S8A provides VE estimates for the primary series per year and age group per scenario.

Vaccine effectiveness is calculated for the primary pertussis vaccination series for children aged 1,2 and 3 years and for the booster dose for those aged 4 to 11 years (with birth cohort ≥ 2005). The error bars indicate the 95% confidence interval.

The VE against notification of pertussis infection of the booster dose with disease onset between 2012 and 2024 was 94% (95% CI: 92–95) at 4 years and 92% (95% CI: 91–94) at 5 years of age, and decreased to 85% (95% CI: 83–87) at 7 years, 71% (95% CI: 66–74) at 9 years and 62% (95% CI: 56–67) at 11 years of age. Supplementary Table S7 provides VE estimates per year and age group. A sensitivity analysis with an 8-percentage-point increase in vaccination coverage for birth cohorts affected by the informed consent procedure shows a small increase of VE (1–6 percentage points). Supplementary Table S8B provides VE estimates for the booster dose per year and age group per scenario.

A sensitivity analysis in which all cases with unknown vaccination status were classified as vaccinated in 2023–24, representing the most conservative VE estimates, resulted in slightly lower VE estimates (2–4 percentage points) for children aged 1, 2, and 3 years. For the booster dose, VE decreased more for the older age groups with already lower VE, 5 percentage points for children aged 4 years and 25% for children aged 11 years. Supplementary Table S9 provides VE estimates per year and age group for the original and most conservative scenario.

The VE against hospitalisation caused by pertussis for the complete primary series was consistently high with 99% (95% CI: 98–99) at 1 year of age, 97% (95% CI: 94–99) at 2 years of age and 93% (95% CI: 83–97) at 3 years of age.

## Discussion

The Netherlands experienced the largest pertussis epidemic since pertussis became notifiable in 1976, with 18,208 notifications in 2024 following a period of very low incidence of notifications between May 2020 and April 2023. Since 2012, the highest incidence of notifications and hospitalisations has consistently been observed in young infants. VE estimates for maternal pertussis vaccination (91%), the primary vaccination series (92–98% for children aged 1 to 3 years) and the booster vaccination (85–94% for children aged 4 to 7 years) remained consistently high with narrow 95% CIs indicating high precision, with no evidence of a decline during the 2023–24 epidemic. Our findings confirm the effectiveness of maternal vaccination and childhood vaccination.

Although pertussis epidemic peaks occur regularly in the Netherlands, the unprecedented surge in 2023–24 is likely attributable to two main factors. Firstly, the disruption of pertussis circulation during the COVID-19 pandemic contributed to reduced acquisition of infection-induced immunity in the population. This was largely due to public health measures such as social distancing and school closures [17-19], thereby increasing the proportion of individuals susceptible to pertussis [20]. Additionally, it is possible that, as a result of COVID-19 measures, individuals with pertussis-like symptoms were less likely to visit their general practitioner. However, this effect is unlikely in infants, given the potentially severe course of pertussis infection in this age group. Secondly, the declining vaccination coverage in the Netherlands [11], together with the known waning of vaccine-derived immunity, particularly among children aged 6–12 years, after the booster dose at 4 years of age [21], probably led to an increased number of susceptible individuals in the Netherlands. Similar surges in pertussis incidence have been reported globally following the pandemic, with many countries reporting sharp increases in pertussis notifications [20,22-25]. In most European Union/European Economic Area (EU/EEA) countries, infants remain the group with the highest reported incidence, although some countries have reported a higher incidence in adolescents aged 10–19 years [24]. These differences may also reflect variations in vaccination schedules, surveillance systems and testing or reporting practices across countries [24]. Testing in infants is likely higher because of the risk of more severe disease compared with older age groups.

Coverage for the DTaP-IPV vaccine in the Netherlands has decreased over time, from 95% in birth cohorts 2005–12 to 93% in the 2019 birth cohort, and further to 89% in the 2022 birth cohort [13]. However, vaccination coverage, particularly from the 2020 birth cohort onwards, may be underestimated as parents have been required to provide informed consent since 2022 to include vaccination status in the national vaccination register [11]. Pijpers et al. identified growing disparities in vaccination uptake in the Netherlands [26]. This is particularly concerning during periods of heightened infection pressure, as observed in 2023–24.

In 2024, during the epidemic, a lower proportion of notified infants were hospitalised compared with previous years. This may have been the effect of increased public and clinical awareness of pertussis during the epidemic, what may have led to earlier healthcare consultations by patients and increased diagnostic testing by healthcare providers. Faster diagnostics could have prevented some hospital admissions. However, it should be noted that hospitalisation information in the notification data is not always accurate. Under-reporting of hospitalisations may have occurred, as hospitalisation data in the notification system may not always be updated after the initial pertussis notification. In general, hospitalisations of infants are probably reported most accurately, since infants are typically admitted to the hospital soon after symptom onset and, in all cases, there is contact with parents to confirm maternal vaccination status. Therefore, notification data may not be the most reliable source for determining the exact number of hospitalisations, but can provide insight into hospitalisation trends over time, with infant hospitalisations more likely reflecting the true incidence than other age groups. In the future, hospitalisation rates may be studied more accurately using electronic health records with ICD-10 coding. However, these data were not yet available for 2023 and 2024 and could not currently be linked to notification data.

Several limitations should be considered when interpreting the findings of this study. Firstly, VE estimates for maternal vaccination, the primary vaccination series, and booster dose were calculated using the screening method, a crude approach that relies on accurate vaccination coverage and routine surveillance data [14]. Our study benefits from the availability of PPV values from the national vaccination register, as reported annually [13]. However, bias could potentially be introduced. For example, mothers who vaccinate might pay more attention to non-pharmaceutical interventions (NPIs), which could have influenced the results, especially during the COVID-19 pandemic when several NPIs were in place. Secondly, the introduction of informed consent in 2022 likely led to an underestimation of vaccination coverage, potentially resulting in underestimated VE [15]. Also, in 2024, a relatively high proportion of cases had an unknown vaccination status due to both the informed consent and incomplete registration in Osiris — though the latter affected only cases and not the background population — which may bias VE estimates upwards. We performed various sensitivity analyses to account for these uncertainties and found that they minimally affected our results. Thirdly, we were unable to include gestational timing and pregnancy duration in our maternal VE estimates, as this information was not available in the notification data. Comparable maternal VE was found in England [27,28], but previous studies have identified that gestational timing (e.g. early vs late in the third trimester) and pregnancy duration (e.g. term vs preterm births) may affect the protective effects of maternal vaccination [29]. This information could improve our VE estimates and provide guidance for optimising maternal vaccination policies. For childhood VE, differences in the type of vaccine and whether maternal vaccination was received, may also be influencing factors [30]. Finally, hospitalisation data may be incomplete because of omissions in updating notifications.

## Conclusion

The Netherlands experienced its largest pertussis epidemic in 2023–24, with the highest incidence of notifications in infants under 6 months. Reduced infection-induced immunity related to the COVID-19 pandemic and waning vaccine-derived immunity in older children, as well as a declining vaccination coverage, likely contributed to the highest pertussis incidence of notifications ever recorded in the Netherlands, affecting all age groups but posing a particular threat to non-immunised infants. Vaccine effectiveness for maternal vaccination, the primary series and booster dose remained consistently high, highlighting the public health priority of enhancing vaccination uptake.

## Data Availability

Data on the aggregated level are available upon request from the corresponding author.

## Supplementary Data

377000 Supplement
