# Supplementary material for: Surveillance and vaccine effectiveness of pertussis, the Netherlands, 2012 to 2024, with an unprecedented surge in 2023 and 2024
Source: Euro Surveill. 2026 Apr 16;31(15):2500919. doi: 10.2807/1560-7917.ES.2026.31.15.2500919 (PMC13090755; doi:10.2807/1560-7917.ES.2026.31.15.2500919)
Supplement: Supplement [file 25-00919_VANROON_Supplement.pdf]

Supplementary files

"This supplementary material is hosted by Eurosurveillance as supporting information alongside the article SURVEILLANCE AND VACCINE EFFECTIVENESS OF PERTUSSIS, THE NETHERLANDS, 2012 TO 2024, WITH AN UNPRECEDENTED SURGE IN 2023 AND 2024, on behalf of the authors, who remain responsible for the accuracy and appropriateness of the content. The same standards for ethics, copyright, attributions and permissions as for the article apply. Supplements are not edited by Eurosurveillance and the journal is not responsible for the maintenance of any links or email addresses provided therein."

Table S1. Vaccination coverage of the Dutch population per birth cohort of the child for the primary pertussis vaccination series, the booster dose and maternal vaccination (13).

Year of birth of the child	Vaccination coverage		
	primary series[#]	booster [*]	Maternal
2004	-	94	-
2005	95	95	-
2006	95	94	-
2007	95	95	-
2008	95	94	-
2009	95	94	-
2010	96	94	-
2011	95	93	-
2012	95	93	-
2013	94	93	-
2014	94	92	-
2015	93	92	-
2016	92	91^	-
2017	93	89^	-
2018	93	82^	-
2019	93^	81^	-
2020	88^	-	70
2021	86^	-	71
2022	89^	-	64^
2023	-	-	64^
2024	-	-	67^

[#] Estimated at 2 years of age, except the estimates for birth cohorts 2019-2021, these are without age limit.

[*] Estimated at 5 years of age, except the estimates for birth cohorts 2016-2018, these are without age limit.

^ The informed consent procedure affects these figures (anonymous vaccinations are excluded; the actual vaccination coverage is likely underestimated).

Table S2. Number of notifications and incidence (per 100,000) of pertussis in the Netherlands by year of symptom onset per age group

Year	Notifications	0-5 m	6-11 m	1-4 y	5-9 y	10-19 y	20-59 y	≥60 y	Total
2012	N	194	44	379	1,552	4,172	5,624	1,854	13,819
	Incidence	216	49	51	160	208	62	49	83
2013	N	56	21	137	323	886	1,394	610	3,427
	Incidence	64	24	19	34	44	15	16	20
2014	N	194	65	491	796	2,834	3,449	1,402	9,231
	Incidence	227	76	68	85	141	38	35	55
2015	N	143	34	278	568	1,956	2,558	1,029	6,566
	Incidence	164	39	39	61	97	28	25	39
2016	N	166	53	405	491	1,423	2,211	813	5,562
	Incidence	195	62	57	53	71	24	20	33
2017	N	137	44	223	421	1,300	2,036	765	4,926
	Incidence	159	51	32	45	64	22	18	29
2018	N	133	57	341	433	1,245	1,960	696	4,865
	Incidence	157	67	49	47	62	22	16	28
2019	N	126	61	314	431	1,608	2,730	1,118	6,388
	Incidence	150	72	45	47	80	30	25	37
2020	N	29	13	47	82	231	388	180	970
	Incidence	34	15	7	9	12	4	4	6
2021	N	8	1	8	3	8	34	17	79
	Incidence	10	1	1	0	0	0	0	0
2022	N	10	9	35	14	16	43	15	142
	Incidence	11	10	5	2	1	0	0	1
2023	N	95	65	184	194	1,212	955	239	2,944
	Incidence	113	78	26	22	61	10	5	16
2024	N	471	367	903	938	3,977	8,430	3,122	18,208
	Incidence	573	446	129	104	202	90	64	102

Table S3. Number and incidence (per 100,000) of notifications reporting hospitalisations due to pertussis per year of symptom onset per age group

Year	Hospitalisations	0-5 m	6-11 m	1-4 y	5-9 y	10-19 y	20-59 y	≥60 y	Total
2012	N	119	4	4	1	3	21	15	167
	Incidence	132	4	0	0	0	0	0	1
2013	N	38	6	4	1	2	7	8	66
	Incidence	43	7	0	0	0	0	0	0
2014	N	120	5	10	3	6	13	17	174
	Incidence	140	6	1	0	0	0	0	1
2015	N	107	3	10	2	6	12	10	150
	Incidence	122	3	1	0	0	0	0	1
2016	N	116	3	7	3	4	8	14	155
	Incidence	136	4	1	0	0	0	0	1
2017	N	105	8	10	4	3	13	11	154
	Incidence	122	9	1	0	0	0	0	1
2018	N	86	16	12	5	3	1	8	131
	Incidence	101	19	2	0	0	0	0	1
2019	N	88	14	11	1	3	9	14	140
	Incidence	104	17	2	0	0	0	0	1
2020	N	23	2	3	1	0	3	2	34
	Incidence	27	2	0	0	0	0	0	0
2021	N	7	1	1	1	0	2	1	13
	Incidence	8	1	0	0	0	0	0	0
2022	N	6	5	7	4	0	1	3	26
	Incidence	7	6	1	0	0	0	0	0
2023	N	58	20	15	3	5	10	17	128
	Incidence	69	24	2	0	0	0	0	1
2024	N	250	76	65	7	15	63	87	563
	Incidence	304	92	9	1	1	1	2	3

Table S4. Maternal Tdap vaccination and related information of notified infants aged 0-2 months, per year of symptom onset

Year	Total notified infants	Total notified infants with known Tdap status	Mother received Tdap		Mother received Tdap, baby was born prematurely		Mother received Tdap, but vaccination was less than 2 weeks before baby was born		Unvaccinated mothers of notified infants (%)
			n	unknown	n	unknown	n	unknown	
2020*	8	8	3	0	0	0	0	0	62,5
2021	0	0	0	0	0	0	0	0	-
2022	5	5	3	0	1	0	0	0	40,0
2023	62	61	5	1	0	1	0	1	91,8
2024	273	241	42	32	9	4	1	4	82,6
Total	348	315	53	33	10	5	1	5	83,2

**Infants with symptom onset from 1 April onwards were included.*

Table S5. Maternal pertussis vaccine effectiveness estimates for infants aged 0-2 months per year based on the original analysis (only infants included with a known Tdap status), the most conservative scenario (infants with unknown Tdap status are considered vaccinated) and the most optimistic scenario (infants with unknown Tdap status are considered unvaccinated).

	Original analysis			All unknowns were vaccinated			All unknowns were unvaccinated		
Year	Total	Mother received Tdap	VE (%)	Total	Mother received Tdap	VE (%)	Total	Mother received Tdap	VE (%)
2020	8	3	74 (-8 – 94)	8	3	74 (-8-94)	8	3	74 (-8-94)
2021	0	0	NA	0	0	NA	0	0	NA
2022	5	3	36 (-285 – 89)	5	3	36 (-285-89)	5	3	36 (-285-89)
2023	62	5	96 (90-98)	62	6	95 (89-98)	62	5	96 (91-98)
2024	241	42	91 (87-94)	273	74	84 (79-88)	273	42	92 (89-94)
Total	315	53	91 (88-94)	348	86	86 (82-89)	348	53	92 (90-94)

Table S6. Pertussis vaccination status of notified cases per year of symptom onset and age group based on the NIP vaccination schedule per birthyear *

Year	Age	Unvaccinated	Partially vaccinated	Fully vaccinated	Unknown	Total known vaccination status	Total	% fully vaccinated
2012	0-3 m	101	0	57	1	158	159	36
	4-5 m	10	4	20	0	34	34	59
	6-11 m	13	1	29	1	43	44	67
	1-4 y	121	11	231	16	363	379	64
	5-8 y	58	206	617	61	881	942	70
	9-11 y	19	162	1422	86	1603	1689	89
	>= 12 y	238	162	4204	5967	4604	10571	91
2013	0-3 m	25	0	20	0	45	45	44
	4-5 m	4	0	6	0	10	10	60
	6-11 m	11	0	10	0	21	21	48
	1-4 y	45	4	87	1	136	137	64
	5-8 y	28	31	107	14	166	180	64
	9-11 y	10	56	326	33	392	425	83
	>= 12 y	36	44	850	1678	930	2608	91
2014	0-3 m	81	0	72	2	153	155	47
	4-5 m	15	1	20	0	36	36	56
	6-11 m	23	1	40	1	64	65	62
	1-4 y	145	15	327	4	487	491	67
	5-8 y	71	101	316	38	488	526	65
	9-11 y	22	223	1086	96	1331	1427	82
	>= 12 y	106	124	2239	4059	2469	6528	91
2015	0-3 m	80	0	45	0	125	125	36
	4-5 m	5	0	12	0	17	17	71
	6-11 m	20	0	14	0	34	34	41
	1-4 y	97	10	169	2	276	278	61
	5-8 y	41	26	309	18	376	394	82
	9-11 y	12	77	697	66	786	852	89
	>= 12 y	80	129	1620	3036	1829	4865	89
2016	0-3 m	94	0	45	1	139	140	32
	4-5 m	15	1	10	0	26	26	38
	6-11 m	29	1	21	2	51	53	41
	1-4 y	199	12	186	8	397	405	47
	5-8 y	49	18	244	28	311	339	78
	9-11 y	16	27	432	30	475	505	91
	>= 12 y	74	103	1294	2623	1471	4094	88
2017	0-3 m	85	0	28	0	113	113	25
	4-5 m	11	5	8	0	24	24	33
	6-11 m	24	1	19	0	44	44	43
	1-4 y	97	12	109	5	218	223	50
	5-8 y	32	16	193	28	241	269	80
	9-11 y	11	29	390	32	430	462	91
	>= 12 y	57	97	1089	2548	1243	3791	88
2018	0-3 m	70	0	41	1	111	112	37

	4-5 m	14	0	6	0	20	20	30
	6-11 m	41	1	15	0	57	57	26
	1-4 y	210	13	113	5	336	341	34
	5-8 y	63	19	188	31	270	301	70
	9-11 y	16	18	441	42	475	517	93
	>= 12 y	52	84	941	2439	1077	3516	87
2019	0-3 m	61	0	43	1	104	105	41
	4-5 m	12	1	8	0	21	21	38
	6-11 m	36	1	22	2	59	61	37
	1-4 y	155	14	133	12	302	314	44
	5-8 y	41	26	193	28	260	288	74
	9-11 y	12	43	495	38	550	588	90
	>= 12 y	47	79	1045	3840	1171	5011	89
2020	0-3 m	15	0	9	0	24	24	38
	4-5 m	2	0	3	0	5	5	60
	6-11 m	7	1	5	0	13	13	38
	1-4 y	26	2	19	0	47	47	40
	5-8 y	13	6	30	6	49	55	61
	9-11 y	4	8	61	1	73	74	84
	>= 12 y	15	19	144	574	178	752	81
2021	0-3 m	4	0	3	0	7	7	43
	4-5 m	0	0	1	0	1	1	100
	6-11 m	0	0	1	0	1	1	100
	1-4 y	0	0	8	0	8	8	100
	5-8 y	0	0	1	1	1	2	100
	9-11 y	0	1	1	0	2	2	50
	>= 12 y	2	0	7	49	9	58	78
2022	0-3 m	1	0	5	0	6	6	83
	4-5 m	1	0	2	1	3	4	67
	6-11 m	2	0	6	1	8	9	75
	1-4 y	5	5	25	0	35	35	71
	5-8 y	0	3	9	0	12	12	75
	9-11 y	0	1	3	0	4	4	75
	>= 12 y	7	4	14	47	25	72	56
2023	0-3 m	62	0	13	1	75	76	17
	4-5 m	15	1	3	0	19	19	16
	6-11 m	52	3	5	5	60	65	8
	1-4 y	154	2	22	6	178	184	12
	5-8 y	64	6	48	16	118	134	41
	9-11 y	21	20	249	16	290	306	86
	>= 12 y	62	88	526	1484	676	2160	78
2024	0-3 m	183	0	109	46	292	338	37
	4-5 m	82	1	22	28	105	133	21
	6-11 m	225	21	34	87	280	367	12
	1-4 y	617	22	135	129	774	903	17
	5-8 y	151	49	348	52	548	600	64
	9-11 y	47	100	1048	54	1195	1249	88
	>= 12 y	59	46	475	14038	580	14618	82

* As only month of birth (for children up to 2 years of age) and the birth year (for all cases) are available, vaccination status was assigned based on the minimum age that the child had definitely reached. For example, if a reported case was three months old (with the birth date always assigned as the 15th of the month) and born in 2024, the child was considered fully vaccinated if its mother had received maternal vaccination or the infant had received at least one dose. This approach was chosen because the child could still be as young as 2.5 months (if the real birthday was on the 30th of the month), and therefore not yet eligible for vaccination at 3 months. Children with a known vaccination status who received less than the recommended vaccinations for their age, were considered partially vaccinated or unvaccinated when they received no vaccinations. When it is unknown if the child and/or the mother received any vaccinations, the vaccination status is "unknown".

Table S7. Vaccine effectiveness of the primary pertussis vaccination series estimated for 1-, 2- and 3-year-olds and the booster dose estimated for 4- to 11-year-olds per year, overall (2012-2024) and divided in the period before the recent outbreak (2012-2022) and the most recent outbreak (2023-2024)

Age in years	1			2			3			4			5			6		
Year	VE (%)	Lower limit 95% CI	Upper limit 95% CI	VE (%)	Lower limit 95% CI	Upper limit 95% CI	VE (%)	Lower limit 95% CI	Upper limit 95% CI	VE (%)	Lower limit 95% CI	Upper limit 95% CI	VE (%)	Lower limit 95% CI	Upper limit 95% CI	VE (%)	Lower limit 95% CI	Upper limit 95% CI
2012	96	94	98	93	89	95	88	81	92	96	93	97	91	86	95	95	93	97
2013	96	91	98	94	88	97	90	80	95	86	53	96	94	89	97	87	74	94
2014	93	89	95	88	82	92	90	86	93	90	83	94	94	91	96	92	87	95
2015	93	89	96	91	85	94	87	78	92	91	82	96	88	80	93	84	74	91
2016	98	97	99	92	88	95	93	90	95	96	93	98	94	90	96	92	86	95
2017	97	94	98	94	89	96	88	80	93	92	84	96	91	83	95	84	71	91
2018	98	96	99	96	93	97	94	91	96	98	95	99	95	92	97	90	83	94
2019	97	96	98	91	87	95	93	90	95	94	88	97	93	87	96	90	84	94
2020	98	92	100	89	68	96	96	88	99	94	65	99	93	78	97	91	78	96
2021	-	-Inf	100	-	-Inf	100	-	-Inf	100	-	-Inf	100	100	-Inf	100	-	-	-
2022	73	-11	94	71	-45	94	81	3	96	-	-Inf	100	77	-154	98	81	-109	98
2023	100	99	100	97	93	99	93	85	97	100	-Inf	100	95	87	98	96	91	98
2024	99	99	99	97	96	98	96	94	97	95	91	97	88	83	92	89	84	93
2012-2024	98	97	98	94	93	94	92	91	93	94	92	95	92	91	94	91	89	92
2012-2022	96	96	97	92	91	93	91	90	92	94	92	95	93	92	94	91	89	92
2023-2024	99	99	99	97	96	98	96	94	97	95	91	97	90	86	93	91	88	94

Age in years	7			8			9			10			11		
Year	VE (%)	Lower limit 95% CI	Upper limit 95% CI	VE (%)	Lower limit 95% CI	Upper limit 95% CI	VE (%)	Lower limit 95% CI	Upper limit 95% CI	VE (%)	Lower limit 95% CI	Upper limit 95% CI	VE (%)	Lower limit 95% CI	Upper limit 95% CI
2012	89	79	94	-	-	-	-	-	-	-	-	-	-	-	-
2013	92	86	96	93	85	97	-	-	-	-	-	-	-	-	-
2014	91	87	94	88	84	91	82	72	89	-	-	-	-	-	-
2015	66	44	80	72	57	82	72	58	81	74	49	87	-	-	-
2016	63	32	80	72	56	82	66	46	78	69	52	80	75	53	87
2017	89	80	93	74	60	83	71	56	81	67	48	79	68	50	79
2018	86	78	92	78	64	86	74	60	83	40	-1	64	71	58	80
2019	73	56	84	83	74	89	67	48	79	65	49	76	71	59	79
2020	77	-12	95	92	79	97	78	45	91	74	24	91	49	-70	85
2021	-	-	-	-	-Inf	100	-	-Inf	100	100	-Inf	100	-	-	-
2022	65	-211	96	-	-Inf	100	-	-Inf	100	-	-Inf	100	100	-Inf	100
2023	93	86	97	91	85	95	83	71	90	49	10	71	63	42	76
2024	87	82	90	72	62	79	62	50	71	67	58	75	46	29	58
2012-2024	85	83	87	80	78	83	71	66	74	64	58	69	62	56	67
2012-2022	84	81	86	81	78	84	72	67	77	64	56	71	70	64	76
2023-2024	88	84	91	78	71	83	67	58	74	64	55	72	50	38	60

Table S8 Sensitivity analysis on the vaccine effectiveness of the primary pertussis vaccination series estimated for 1-, 2- and 3-year-olds and the booster dose estimated for 4- to 6-year-olds overall (2012-2024) and divided in the period before the recent outbreak (2012-2022) and the most recent outbreak (2023-2024) with a vaccination coverage of the affected birth cohorts (2021 and 2022 for the primary series and 2018 and 2019 for the booster dose) increased with 3% or 8% for the primary series and booster dose, respectively.

A. Primary series*

Age (yr)	1						2						3					
Year	Original			Scenario			Original			Scenario			Original			Scenario		
	VE	LL	UL	VE	LL	UL	VE	LL	UL	VE	LL	UL	VE	LL	UL	VE	LL	UL
2022	73	-11	94	79	13	95												
2023	100	99	100	100	99	100	97	93	99	98	95	99						
2024	99	99	99	99	99	100	97	96	98	98	97	98	96	94	97	96	94	97
2012-2024	98	97	98	98	97	98	94	93	94	94	93	95	92	91	93	92	91	93
2012-2022	96	96	97	96	96	97												
2023-2024	99	99	99	99	99	100	97	96	98	98	97	98	96	94	97	96	94	97

* VE is not shown for year-age combinations that were not affected by the change in vaccination coverage because children born in 2021 and 2022 have not reached this age yet.

B. Booster dose

Age (yr)	4						5						6					
	Original			Scenario			Original			Scenario			Original			Scenario		
Year	VE	LL	UL	VE	LL	UL	VE	LL	UL	VE	LL	UL	VE	LL	UL	VE	LL	UL
2022	-	-Inf	100	-	-Inf	100	77	-154	98	77	-154	98	81	-109	98	81	-109	98
2023	100	-Inf	100	100	-Inf	100	95	87	98	97	93	99	96	91	98	96	91	98
2024	95	91	97	97	95	98	88	83	92	94	91	96	89	84	93	90	85	93
2012-2024	94	92	95	94	93	95	92	91	94	93	92	94	91	89	92	91	89	92
2012-2022	94	92	95	94	92	95	93	92	94	93	92	94	91	89	92	91	89	92
2023-2024	95	91	97	97	95	98	90	86	93	95	93	96	91	88	94	92	88	94

Table S9. Sensitivity analysis on the vaccine effectiveness of the primary pertussis vaccination series estimated for 1-, 2- and 3-year-olds and the booster dose estimated for 4- to 11-year-olds overall (2012-2024) and divided in the period before the recent outbreak (2012-2022) and the most recent outbreak (2023-2024) in which all cases with unknown vaccination status were classified as vaccinated, representing the most conservative estimates.

Age (yr)	1						2						3						4						5						6					
	Original			Scenario			Original			Scenario			Original			Scenario			Original			Scenario			Original			Scenario			Original			Scenario		
Year	VE	LL	UL	VE	LL	UL	VE	LL	UL	VE	LL	UL	VE	LL	UL	VE	LL	UL	VE	LL	UL	VE	LL	UL	VE	LL	UL	VE	LL	UL	VE	LL	UL	VE	LL	UL
2012-2024	98	97	98	96	96	97	94	93	94	92	91	93	92	91	93	91	89	92	94	92	95	93	91	94	92	91	94	89	87	90	91	89	92	86	84	88
2012-2022	96	96	97	95	94	96	92	91	93	92	90	93	91	90	92	89	88	90	94	92	95	93	91	94	93	92	94	90	88	92	91	89	92	87	84	89
2023-2024	99	99	99	97	96	98	97	96	98	94	92	95	96	94	97	92	89	94	95	91	97	90	85	94	90	86	93	82	75	87	91	88	94	86	81	90

Age (yr)	7						8						9						10						11					
	Original			Scenario			Original			Scenario			Original			Scenario			Original			Scenario			Original			Scenario		
Year	VE	LL	UL	VE	LL	UL	VE	LL	UL	VE	LL	UL	VE	LL	UL	VE	LL	UL	VE	LL	UL	VE	LL	UL	VE	LL	UL	VE	LL	UL
2012-2024	85	83	87	77	73	81	80	78	83	70	65	74	71	66	74	50	40	58	64	58	69	39	27	50	62	56	67	31	17	43
2012-2022	84	81	86	75	70	80	81	78	84	70	64	75	72	67	77	50	37	59	64	56	71	24	0	42	70	64	76	37	18	52
2023-2024	88	84	91	82	76	87	78	71	83	69	59	77	67	58	74	50	34	62	64	55	72	52	38	63	50	38	60	25	2	43
